# Short-channel regression in functional near-infrared spectroscopy is more effective when considering heterogeneous scalp hemodynamics

**DOI:** 10.1117/1.NPh.7.3.035011

**Published:** 2020-09-29

**Authors:** Dominik Wyser, Michelle Mattille, Martin Wolf, Olivier Lambercy, Felix Scholkmann, Roger Gassert

**Affiliations:** a ETH Zurich, Department of Health Sciences and Technology, Rehabilitation Engineering Laboratory, Zurich, Switzerland; b University Hospital Zurich, University of Zurich, Department of Neonatology, Biomedical Optics Research Laboratory, Zurich, Switzerland; c University of Bern, Institute of Complementary and Integrative Medicine, Bern, Switzerland

**Keywords:** functional near-infrared spectroscopy, short-channel regression, scalp hemodynamics, brain–computer interface, physiological systemic artifacts, general linear model

## Abstract

**Significance:** The reliability of functional near-infrared spectroscopy (fNIRS) measurements is reduced by systemic physiology. Short-channel regression algorithms aim at removing systemic “noise” by subtracting the signal measured at a short source–detector separation (mainly scalp hemodynamics) from the one of a long separation (brain and scalp hemodynamics). In literature, incongruent approaches on the selection of the optimal regressor signal are reported based on different assumptions on scalp hemodynamics properties.

**Aim:** We investigated the spatial and temporal distribution of scalp hemodynamics over the sensorimotor cortex and evaluated its influence on the effectiveness of short-channel regressions.

**Approach:** We performed hand-grasping and resting-state experiments with five subjects, measuring with 16 optodes over sensorimotor areas, including eight 8-mm channels. We performed detailed correlation analyses of scalp hemodynamics and evaluated 180 hand-grasping and 270 simulated (overlaid on resting-state measurements) trials. Five short-channel regressor combinations were implemented with general linear models. Three were chosen according to literature, and two were proposed based on additional physiological assumptions [considering multiple short channels and their Mayer wave (MW) oscillations].

**Results:** We found heterogeneous hemodynamics in the scalp, coming on top of a global close-to-homogeneous behavior (correlation 0.69 to 0.92). The results further demonstrate that short-channel regression always improves brain activity estimates but that better results are obtained when heterogeneity is assumed. In particular, we highlight that short-channel regression is more effective when combining multiple scalp regressors and when MWs are additionally included.

**Conclusion:** We shed light on the selection of optimal regressor signals for improving the removal of systemic physiological artifacts in fNIRS. We conclude that short-channel regression is most effective when assuming heterogeneous hemodynamics, in particular when combining spatial- and frequency-specific information. A better understanding of scalp hemodynamics and more effective short-channel regression will promote more accurate assessments of functional brain activity in clinical and research settings.

## 1 Introduction

Functional near-infrared spectroscopy (fNIRS) enables the noninvasive measurement of human brain activity by monitoring concentration changes of oxygenated hemoglobin ($O_{2}\mathrm{Hb}$) and deoxygenated hemoglobin (HHb) in the blood.^1^^–^^4^ fNIRS has evolved from a tool for basic research to a widely used technique to investigate brain activity in nonconstrained environments.^5^^,^^6^ Despite its versatile use, there remain several challenges, in particular, the sensitivity of continuous-wave fNIRS to hemodynamic changes of non-neuronal origin.^2^^,^^7^^–^^10^ These are often referred to as physiological “noise” or “interference” and include systemic activities, such as cardiac pulsation (1 to 2 Hz), respiration (0.2 to 0.4 Hz), low-frequency oscillations (∼0.1  Hz) and very low-frequency oscillations (0.01 to 0.05 Hz),^11^ and an increase in blood flow through sympathetic nervous activity.^12^ These artifacts generate signal changes that may mimic or mask true task-evoked hemodynamic responses (HRs) and may lead to false positives or false negatives.^8^^,^^10^^,^^13^ This challenge has been acknowledged and its significance recognized in the recent years by the fNIRS community.^8^ Although the susceptibility to non-neuronal signals is specific to the measurement principle of fNIRS, all technologies that infer brain activity via hemodynamic changes, i.e., fNIRS, functional magnetic resonance imaging, and positron emission tomography, are affected.

As a main contributor to low-frequency oscillations, Mayer waves (MW) are rhythmic hemodynamic oscillations in arterial blood pressure,^14^ and are presumably the main reason why it is not possible to recover a functional HR in some subjects.^15^ Cardiac and respiratory signals can be removed with low-pass filters, when adequately selected for the specific measurement protocols and task/stimulus durations.^16^^,^^17^ The removal of the other systemic signals is more difficult and requires the application of more elaborate signal processing since their frequency contents overlap with the functional HR.^18^^–^^20^ Short-channel regression methods have been proposed as a means to separate cerebral from systemic activity.^21^^,^^22^ Through the separate measurement of the scalp hemodynamics by means of a short-separation (SS) channel (typically <15  mm and ideally 8.4-mm length^23^^,^^24^), a signal that predominantly contains systemic and minimal brain activity is obtained. To extract the contribution of the brain from a long-separation (LS) fNIRS measurement (typically 30 mm), the SS is subtracted from the LS signal. Short-channel regression has been shown to significantly improve the quality of the recovered functional brain activity.^18^^,^^21^^,^^22^^,^^25^

However, conflicting approaches on how to apply these scalp regressors can be found in the literature, especially, on the assumed spatial distribution of the scalp hemodynamics, and there is currently no consensus on which approach is better. Systemic artifacts are typically not constrained locally, but they affect the whole brain and extracerebral tissues, and are thus considered “global.” As a consequence, it is often assumed that global noise is distributed homogeneously over the entire scalp layer and thus that a single global signal can be used for the short-channel regression.^26^^–^^30^ In contrast, it was repeatedly shown that scalp hemodynamics follow spatially heterogeneous patterns,^9^^,^^17^^,^^31^^–^^38^ i.e., different short-channel signals are measured at different locations on the scalp. An additional complexity is observed when considering that the physiological artifacts also show frequency-dependent spatial variations,^36^ with a noteworthy effect observed for the MW-frequency band (i.e., average time lags up to 2 s between ipsilateral head regions^36^). It is not entirely understood yet how the scalp hemodynamics behave spatially and how strongly their frequency-specific behavior affects the effectiveness of short-channel regression, raising the question of how to optimally apply short-channel regressors.

In this work, we investigate the scalp hemodynamics above sensorimotor areas with respect to the two differing assumptions of their spatial distribution (heterogeneous versus homogeneous) and their effectiveness for short-channel regression. Through the combination of local and global regressors, we strive to regress systemic signals of cerebral and extracerebral tissue origin more completely. We further hypothesize that the separate consideration of temporal heterogeneities in form of MW oscillations enables a more optimal regression of systemic signals by reducing the physiological noise. To do so, we propose an algorithm based on a general linear model (GLM) that makes use of nonlinear least squares. This work is important as it paves the way for the implementation of more accurate short-channel regression that could increase the overall quality of fNIRS measurements, allowing more wide-spread application of this technology. This can particularly help to promote clinical applications by making measurements more reliable at the individual level.

## 2 Methods

To investigate scalp hemodynamics over sensorimotor areas and its influence on the estimation of the functional HR, we conducted an fNIRS experiment to obtain resting-state and motor-execution measurements. The study was conducted in accordance with the Declaration of Helsinki Ethical Principles and Good Clinical Practices, and all subjects provided informed consent.

### 2.1 Subjects and Data Acquisition

**Fig. 1:**
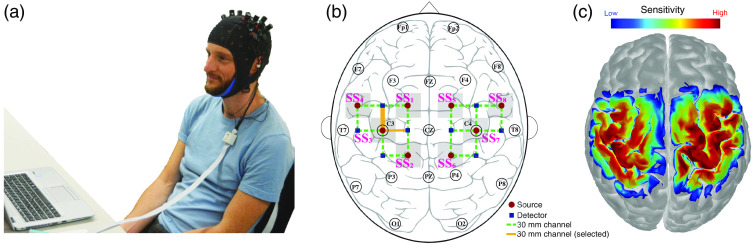
Experimental setup. (a) Subjects were seated in front of a screen with the NIRSport system mounted. (b) Optode arrangement on the head. Gray areas indicate the locations of the SS channels (${\mathrm{SS}}_{1}$ to ${\mathrm{SS}}_{8}$), dashed green lines the LS channels that were measured, and the two orange lines the selected channels with highest t-values. The selected channel for subject 1, 2, 3, and 5 was anterior to the C3 location (thick orange line), and superior for subject 4 (thin orange line). (c) Sensitivity map of the measurement arrangement.

Five male, right-handed volunteers (aged 27±4.7 years, range: 24 to 35 years) participated in the experiment. Subjects were seated in front of a computer screen in a dark room, with their hands lying comfortably on their lap [Fig. 1(a)]. The experiment was split into one 15-min resting-state run and two 15-min runs of motor-execution tasks. The resting-state run was motivated by the need for subject-specific rest data to which an artificial HR could later be added for further validation of the regressors. One motor-execution run consisted of 20 block-designed trials each involving 16 s of self-paced opening and closing of the right hand at ∼1  Hz (onscreen instructions: green indicator and command “move hand”), followed by an intertrial time that was randomized to 14, 16, or 18 s (“rest”). The subjects were instructed to perform a smooth grasping movement. Two seconds before trial start, the subjects were informed about the upcoming task (“get ready”) to ensure they were paying attention. During data acquisition, subjects were instructed to sit still and to move only their right hand when the instruction “move hand” was displayed. Subject 5 performed only one motor-execution run due to discomfort.

The measurements were performed with the NIRSport system (NIRx Medizintechnik GmbH, Berlin, Germany). The system measured with eight sources and eight detectors at two wavelengths (760 and 850 nm), with a sampling frequency of 7.8125 Hz. For the conduction of this study, an fNIRS cap enabling the measurement with SS and LS channels was deployed. The cap was provided by the manufacturer and enabled the concomitant measurement with 20 LS channels with a source–detector separation of 30 mm and 8 SS channels of 8-mm distance that were located around each light source [${\mathrm{SS}}_{1}$ to ${\mathrm{SS}}_{8}$ in Fig. 1(b)]. The optodes were placed over the left and right parietal lobes covering the sensorimotor cortex. Two sources (${\mathrm{SS}}_{3}$ and ${\mathrm{SS}}_{7}$) were placed over the left and right primary motor cortices on C3 and C4 according to the international 10–20 system of electrode placement [Fig. 1(b)]. The other optodes were located over the left and right premotor cortices (${\mathrm{SS}}_{4}$ and ${\mathrm{SS}}_{8}$), supplementary motor area (${\mathrm{SS}}_{1}$ and ${\mathrm{SS}}_{5}$), and primary somatosensory cortices (${\mathrm{SS}}_{2}$ and ${\mathrm{SS}}_{6}$). Center-to-center distances^33^ between LS and SS channels were 15, 34, 54, 62, and 75 mm per hemisphere.

### 2.2 Data Processing

Data were processed using custom-built MATLAB scripts (Version 2018b, MathWorks, Massachussets, US). Channels with low signal quality were excluded from data analysis. They were identified based on the evaluation of the cardiac signal by the method of Perdue et al.^39^ More specifically, a Gaussian curve was fitted into the frequency spectrum of $O_{2}\mathrm{Hb}$ between 0.6 and 1.8 Hz, and a peak signal of ≥12  dB was marked as good signal quality. This approach enables the detection of channels that have a strong optical signal but lack of physiological signal content, which may occur in SS channels. For subjects 2 and 3, the channels ${\mathrm{SS}}_{6}$ and ${\mathrm{SS}}_{8}$ were removed due to low signal quality, respectively. ${\mathrm{SS}}_{8}$ was excluded for subject 4 due to repeated data dropout of the corresponding sensor.

Motion artifacts were minimized with a movement artifact reduction algorithm involving detection by moving standard deviation and removal by spline fitting.^40^ After applying the modified Beer–Lambert law,^41^ with differential pathlength factors of 6.1 at 760 nm and 5.6 at 850 nm,^42^^,^^43^ the concentration changes of $O_{2}\mathrm{Hb}$ and HHb were filtered bidirectionally (MATLAB: filtfilt; finite impulse response, order 1000^16^) at two passbands with regard to the signal of interest:^15^^,^^36^ HR band (0.01 to 0.3 Hz) and MW band (0.07 to 0.14 Hz). It is important to note that a narrow definition of the HR band was chosen in this work and little power (<‱) of the HR may exceed this range, as well as that the frequency spectrum will change for study protocols with different block durations. In this work, only $O_{2}\mathrm{Hb}$ was investigated, because it is influenced to a higher degree by confounding factors than HHb.^15^^,^^18^^,^^32^^,^^44^

The LS channel with the strongest cerebral activation during the hand-grasping experiment according to GLM t-statistics (see Sec. 2.5) was selected for the subsequent analysis. For all subjects, the selected channel originated from the source placed over C3 (${\mathrm{SS}}_{2}$). The corresponding detector optode was located anterior for subjects 1, 2, 3, and 5 [thick orange line in Fig. 1(b)], and superior for subject 4 (thin orange line). For all subjects, the channel flagged for the strongest activation was located over the expected primary motor cortex area of the brain.

### 2.3 Short-Channel Regression Using GLM

The GLM method is a commonly known technique that allows the regression of short channels and that is relatively easy to implement.^12^^,^^38^^,^^45^^–^^47^ It is known that the regression of different regressor signals may alter the shape of the recovered signal.^36^ Therefore, we applied five regressor sets incorporating different assumptions on the spatial distribution of systemic interference. Three approaches were implemented according to literature and relied on the standard GLM method [i.e., (i) one local regressor, (ii) one global regressor, and (iii) one local and one global regressor], whereas two approaches were based on physiological assumptions and made use of an adapted GLM method using non-negative least squares [i.e., (iv) one local, one global and one MW-bandpass filtered global regressor, and (v) all available SS channels and their MW-bandpass filtered signal].

#### 2.3.1 Classical GLM

In GLM, the measurements (Y), the explanatory variables (X), and the error term (ε) are linked to each other according to

Y=Xβ+ε, (1)

where β is the model parameters/weights. The design matrix X consists of a constant-offset array ($X_{C}$), a modeled HR ($X_{\mathrm{HR}}$), and a nuisance regressor from the superficial scalp layer ($X_{\mathrm{SCALP}}$)

$$X=[X_{C},X_{\mathrm{HR}},X_{\mathrm{SCALP}}].$$ (2)

To solve Eq. (1) and obtain an estimated parameter β^, ordinary least squares using the Moore–Penrose pseudoinverse is applied

β^=(X⊤X)−1X⊤Y. (3)

An estimated, cerebral activity y^C is obtained after subtraction of XSCALPβ^SCALP from an LS measurement $y_{\mathrm{LS}}$.

Three approaches based on literature used the standard GLM method: (i) selecting a local regressor ($X_{\mathrm{SCALP}}=X_{\mathrm{SS}}$) from the most proximal SS channel ${\mathrm{SS}}_{3}$ (method: ${\mathrm{GLM}}^{\mathrm{SS}}$),^18^ (ii) using a global regressor ($X_{\mathrm{SCALP}}≔X_{\mathrm{PCA}}$) derived from the first principal component after principal component analysis (PCA) of all SS channels (method: ${\mathrm{GLM}}^{\mathrm{PCA}}$),^29^ and (iii) the combination of the local and the global PCA regressor ($X_{\mathrm{SCALP}}=[X_{\mathrm{SS}},X_{\mathrm{PCA}}]$) to assume separate superficial and global brain noise (method: ${\mathrm{GLM}}^{\mathrm{SS}+\mathrm{PCA}}$).^17^

#### 2.3.2 Non-negative GLM

**Fig. 2:**
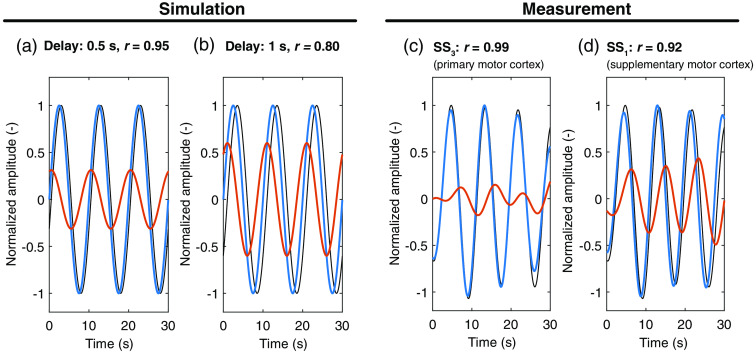
Simulations and sample signals of MWs. Subtraction of two signals in the MW band using least-squares minimization. The black line shows the reference signal, the blue line shows the regressor signal, and the red line shows the residual after subtraction of the other two signals. (a) and (b) Simulated signals at 0.1 Hz with time lags of 0.5 and 1 s between reference and regressor signal, respectively. The regressed signal (red line) has residual magnitudes of 30% and 60% of the original signals. (c) and (d) Example of a MW-bandpass filtered $O_{2}\mathrm{Hb}$ measurement, showing one LS channel over the primary motor cortex (${\mathrm{SS}}_{3}$, black line) in comparison to a SS channel located over the primary motor cortex (${\mathrm{SS}}_{4}$, blue line) or supplementary motor area. The regressed signal (red line) shows the influence of location-dependent phase shift.

The two other GLM methods were based on the assumption of phase-shifted oscillations in the MW band.^12^ This facilitates the reduction of residuals, as shown in Fig. 2.Two sinusoidal oscillations at 0.1 Hz with time lags of 0.5 s [Pearson’s r=0.95, Fig. 2(a)] and 1 s [r=0.80, Fig. 2(b)] are represented, respectively, to simulate two phase-shifted MW signals. After regression,^21^ the introduced time lags lead to new oscillations with residual magnitudes of 30% and 60% of the original signal. It is worthy to note that the residuals would have had zero magnitude without the presence of phase shift. In fNIRS signals, similar delays for the MW oscillations are observed^36^ [Figs. 2(c) and 2(d)].

Non-negative least squares^48^^–^^50^ was applied for the estimation of β^ to reduce the risk of overfitting when including additional regressors. Non-negative least squares (MATLAB: lsqnonneg) iteratively minimizes the least-squared error between observation and the expected values by setting negative values to 0.^51^ It prevents the arbitrary combination of multiple regressors by forcing only positive linear combinations of physiological regressor signals. This approach is based on the physical foundation that concentration changes measured by fNIRS must be a summation of physiological signals originating from cortical and noncortical regions.^21^ Conceptually, this reflects the fact that a regressor (e.g., scalp signal) is not able to “generate” photons but only to absorb them. Applying non-negative least squares is not a necessity when considering multiple (phase shifted) regressors and similar results may be obtained with classical GLM, but the constraints preclude unrealistic outcomes of GLM regression.^52^ Equation (1) is then redefined as

Y=Xβ+εsubject to  β≥0. (4)

To prevent a biased estimate by restricting $X_{C}$ and $X_{\mathrm{HR}}$ to be multiplied with positive values only, X was extended with their negative duplication

$$X=[\pm X_{C},\pm X_{\mathrm{HR}},+X_{\mathrm{SCALP}}].$$ (5)

**Fig. 3:**
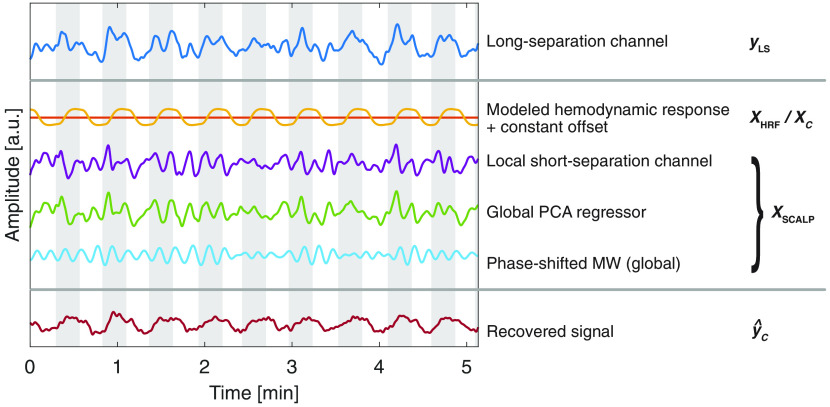
Example of GLM regression. The time course of an LS signal $y_{\mathrm{LS}}$ (only $O_{2}\mathrm{Hb}$ was investigated in this work) is regressed with the GLM approach (the $nn{\mathrm{GLM}}^{\mathrm{SS}+\mathrm{PCA}}$ method is shown as an example). The design matrix X consists of a constant offset $X_{C}$, a model of the HR function ($X_{\mathrm{HR}}$, the time and dispersion derivatives are not shown), and the scalp regressors $X_{\mathrm{SCALP}}$. The estimated cerebral activity y^C is obtained by performing least-squares minimization and subtracting $X_{\mathrm{SCALP}}$ from $y_{\mathrm{LS}}$. The shaded areas represent the hand-grasping trials, whereas the white areas represent rest phases.

The fourth (iv) GLM approach (method: $nn{\mathrm{GLM}}^{\mathrm{SS}+\mathrm{PCA}}$, exemplarily shown in Fig. 3) is an extension of ${\mathrm{GLM}}^{\mathrm{SS}+\mathrm{PCA}}$ with a third scalp signal being the phase-shifted PCA-MW signal $X_{\mathrm{PCA}}^{\mathrm{MW},\mathrm{PS}}$ ($X_{\mathrm{SCALP}}=[X_{\mathrm{SS}},X_{\mathrm{PCA}},X_{\mathrm{PCA}}^{\mathrm{MW},\mathrm{PS}}]$). This approach considers delays in the MW band between the global component and the LS signal. The $X_{\mathrm{PCA}}^{\mathrm{MW},\mathrm{PS}}$ was obtained as follows: first, $X_{\mathrm{PCA}}$ and $y_{\mathrm{LS}}$ were bandpass filtered in the MW band to obtain $X_{\mathrm{PCA}}^{\mathrm{MW}}$ and $y_{\mathrm{LS}}^{\mathrm{MW}}$. Second, $X_{\mathrm{PCA}}^{\mathrm{MW}}$ was phase shifted in the range of ±3  s. Third, ordinary least squares was iteratively applied for the three bandpass-filtered signals, $y_{\mathrm{LS}}^{\mathrm{MW}}$, $X_{\mathrm{PCA}}^{\mathrm{MW}}$, and phase-shifted $X_{\mathrm{PCA}}^{\mathrm{MW}}$ to find the optimally delayed signal $X_{\mathrm{PCA}}^{\mathrm{MW},\mathrm{PS}}$ that minimizes the residual errors.

The fifth (v) approach (method: $nn{\mathrm{GLM}}^{\mathrm{multiSS}}$) was an attempt to minimize systemic interference by including all spatial and temporal information available from the scalp measurements. It was not based on an underlying physiological model but was an approach to extract maximal information available from the superficial layer. The scalp regressor matrix was constructed from all SS channels $X_{\mathrm{multiSS}}$ and their phase-shifted MW signal $X_{\mathrm{multiSS}}^{\mathrm{MW},\mathrm{PS}}$, obtained the same way as described in the previous paragraph for (iv) ($X_{\mathrm{SCALP}}=[X_{\mathrm{multiSS}},X_{\mathrm{multiSS}}^{\mathrm{MW},\mathrm{PS}}]$).

### 2.4 Simulation of Hemodynamic Response Time Series

A major challenge when analyzing fNIRS measurements is that no ground truth for neuronal activity is available. Therefore, we performed simulations where a blocked design of two conditions (task and rest) convolved with the HR function was superimposed on the measurements from the resting-state run.^22^ The double gamma kernel had an amplitude of 0.3  μM for $O_{2}\mathrm{Hb}$,^18^ an onset delay of 0.1 s, and a time-to-peak of 6.7 s.^53^ For each of the 5 subjects, 18 trials of 16 s were simulated and superimposed on the LS channel located over C3, and intertrial times were randomized between 14 and 18 s identical to the motor-execution run. The simulations were repeated three times with randomized intertrial durations. In total, 270 trials were simulated.

We deliberately used real resting-state measurements instead of purely simulated systemic signals, because we expected that simulated noise could only insufficiently reflect heterogeneous behavior in the scalp and brain layers. A possible limitation of this approach is that resting-state measurements may contain spontaneous neural activity with amplitudes comparable to functional brain activity,^7^^,^^54^^,^^55^ hampering the efficacy of the regression. Therefore, it is important to investigate the performance of the different GLM approaches through simulations and actual motor-execution runs.

### 2.5 Data Analysis

The normalized MW amplitude $A_{\mathrm{MW}}^{N}$ was calculated as the ratio of the amplitudes of the MW oscillations divided by the amplitude of the cardiac signal. These signal amplitudes were obtained with the square root of the signal power (MATLAB: bandpower) for the frequency ranges of 0.07 to 0.14 Hz and 0.6 to 2 Hz, respectively. For every subject, the median value of all LS measurements with high signal quality (a Gaussian peak fit with strength above 10 dB^39^) served as representative value.

Pearson’s correlation coefficient (r) was calculated for all combinations of the 8 SS channels and the selected LS channel. For the subjects with two runs (subjects 1 to 4), the mean of both runs was calculated after applying Fisher z-transformation to compensate for skewness effects, followed by backward transformation.^18^ Interchannel time lags were obtained from cross-correlation analysis with maximal time lags of ±5  s.

For evaluation of the regression results, GLM was applied on all regressed signals. The GLM approach as shown in Eqs. (1) and (3) was used, with the design matrix X consisting only of $X_{C}$ and $X_{\mathrm{HR}}$ (i.e., $X_{\mathrm{SCALP}}≔0$). In this work, we tested the null hypothesis that during a trial the recovered LS signal did not change.^56^ From the solution of the GLM model, we calculated for every run (i) the t-value as t=(c⊤β^)/[var(ε)c⊤(X⊤X)−1c],^57^ with c being the contrast vector, and ε the estimated residuals, (ii) the Pearson’s correlation coefficient (r) between recovered and fitted time courses, and (iii) the root-mean-square error (RMSE) of the residuals. Statistical differences between regression methods were determined using two-tailed, paired t-tests. Fisher z transformation was applied on correlation data prior to the t-test.

To fathom the potential of the regression approaches for protocols that are based on single-trial evaluation, such as may be the case for brain–computer interfaces (BCI), we additionally calculated a trial-based contrast-to-noise ratio (CNR) metric. The results of the CNR evaluation are presented in the Appendix.

## 3 Results

The results section is split into three parts. First, the spatiotemporal distribution of scalp hemodynamics over sensorimotor areas is presented with respect to the incongruent assumptions of heterogeneity and homogeneity (Sec. 3.1). Second, the performance of the five GLM regression approaches is reported for simulated HRs, which were overlaid on actual resting-state measurements (Sec. 3.2). Third, the performance of the same regression methods is presented for a hand-grasping experiment (Sec. 3.3).

### 3.1 Behavior of Scalp Hemodynamics over Sensorimotor Areas

The normalized MW amplitudes ($A_{\mathrm{MW}}^{N}$) for $O_{2}\mathrm{Hb}$ for the resting-state and motor-execution measurements are shown in Table 1, showing that the MW amplitudes varied substantially between subjects. Large MW amplitudes were present for subjects 3 and 5, indicating that it may be more difficult to recover an HR within the strong physiological noise, as compared to the other subjects with weaker MW oscillations.

**Table 1:** Normalized amplitude of MWs for $O_{2}\mathrm{Hb}$. Median normalized amplitude in the MW band ($A_{\mathrm{MW}}^{N}$) for all LS channels with good signal quality for $O_{2}\mathrm{Hb}$. The MW amplitude was normalized using the amplitude of the cardiac signal. In parenthesis, the interquartile ranges are shown.

	Subject 1	Subject 2	Subject 3	Subject 4	Subject 5
Resting state	0.58 (0.52 to 0.61)	0.52 (0.39 to 0.57)	1.18 (0.89 to 1.44)	0.81 (0.71 to 1.03)	1.61 (1.46 to 2.47)
Motor execution	0.54 (0.51 to 0.59)	0.47 (0.39 to 0.64)	1.28 (0.97 to 1.40)	0.79 (0.62 to 1.02)	1.06 (0.92 to 1.48)

**Fig. 4:**
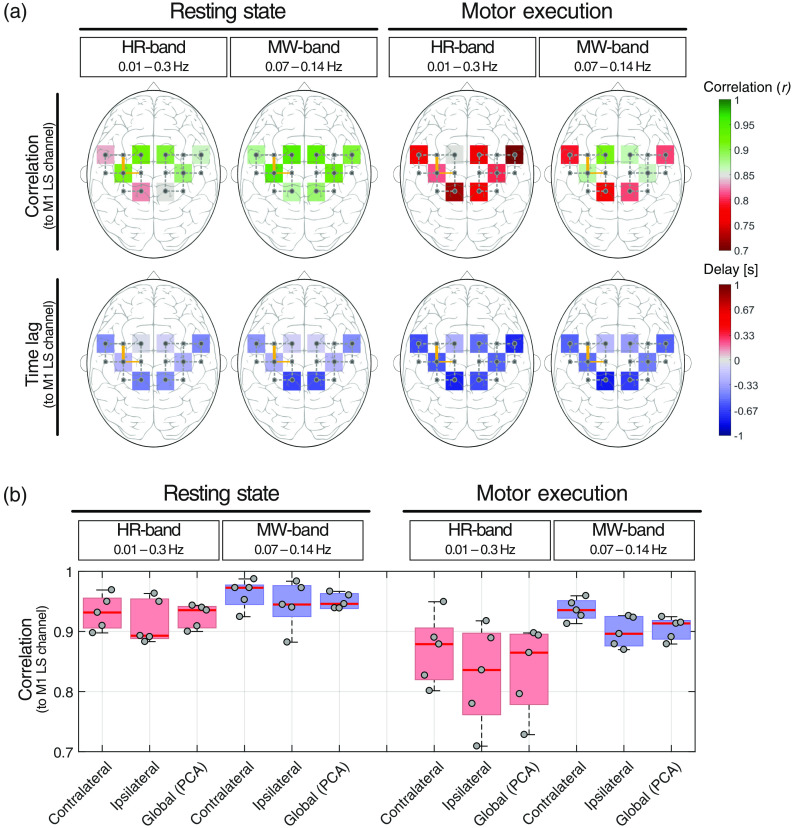
Group-level correlation analysis of SS channels. Group-averaged correlation values and time lags for the resting-state and right-hand grasping experiments. All correlations of the SS channels were calculated with respect to the LS channel placed over the left primary motor cortex (M1, orange lines). (a) The correlations and time lags are projected onto a standard head. The colored squares indicate the location of the SS channels. (b) Correlations for different experimental states (resting state versus motor execution), frequency bands (HR versus MW band) and spatial conditions (ipsilateral, contralateral, and global). For every state, the channel with the highest correlation was selected. The gray dots and red lines indicate individual subjects (subjects 1 to 5 in horizontal order) and the group median values, respectively.

Correlation and phase-shift analysis of the eight SS channels in comparison with a reference LS channel placed over primary motor cortex are shown in Fig. 4(a). For the HR and the MW bands during resting state, globally high Pearson’s r (>0.8) was observed with a weak interhemispheric symmetry, where the SS optodes placed over C3 (i.e., ${\mathrm{SS}}_{1}$ and ${\mathrm{SS}}_{3}$) and C4 (i.e., ${\mathrm{SS}}_{5}$ and ${\mathrm{SS}}_{7}$) were most similar to the LS channel. The symmetric pattern was more pronounced for the motor-execution measurements, with the same short channels (i.e., ${\mathrm{SS}}_{1}$, ${\mathrm{SS}}_{3}$, ${\mathrm{SS}}_{5}$, and ${\mathrm{SS}}_{7}$), being the closest and the symmetrically opposite SS channels to the reference LS channel, exhibiting the highest correlations, and the frontal (i.e., ${\mathrm{SS}}_{5}$ and ${\mathrm{SS}}_{8}$) and occipital (i.e., ${\mathrm{SS}}_{2}$ and ${\mathrm{SS}}_{6}$) channels having lower correlations.

**Fig. 5:**
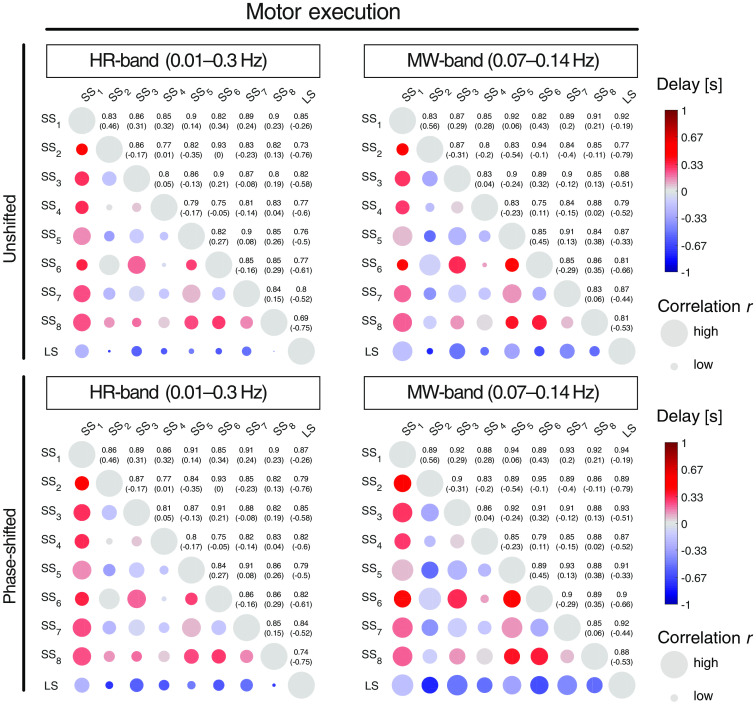
Group-level correlation matrix during hand-grasping experiment. The upper two panels show the unshifted correlation values for all channel-pairs, and the lower two panels were obtained for optimally phase-shifted signals. The diameter of the colored circles corresponds to Pearson’s correlation r: a bigger circle implies a higher correlation (first numbers in the upper-right triangles). The color relates to the time shift in seconds (the lower number in parenthesis): red indicates a positive, blue a negative time lag. A blue circle indicates that the horizontal signal labeled on the left side (row) lags behind the vertical signal labeled on the top (column). ${\mathrm{SS}}_{1}$ to ${\mathrm{SS}}_{8}$ denote the short-separation channels, LS is the long-separation channel placed over C3.

Short channels with lower correlations tended to have larger time lags with respect to the reference LS channel. ${\mathrm{SS}}_{3}$ had an average time lag of 0.2±0.37  s (resting state) and 0.58±0.44  s (motor execution) in the HR band, and 0.25±0.27  s (resting state) and 0.51±0.36  s (motor execution) in the MW band. A delay between the LS and the SS channels was observed for all frequency bands and conditions at a group level. Figure 4(a) graphically shows the last columns of the unshifted correlation matrices for the motor-execution runs shown in Fig. 5 (upper panels).

Correlation values for three different spatial conditions (contralateral, ipsilateral, and global) were investigated for the same frequency bands (i.e., MW and HR bands) and experiments (i.e., resting state and motor execution) as before, and are shown in Fig. 4(b). For the contralateral (i.e., left hemisphere for the right-hand grasping task) and ipsilateral (i.e., right hemisphere for the right-hand grasping task) cases, only the SS channels with the highest correlations to the reference LS channel were used. For the global condition, the correlation between the reference LS channel and the PCA model was calculated. The correlation values were high (r>0.9) during resting state and both frequency bands for all three conditions, with slightly higher values for the contralateral than the ipsilateral or global conditions. The motor-execution experiment exhibited decreased correlation values for all conditions and frequency bands, with a much stronger effect for the HR band than the MW band. Among all conditions, the correlations remained relatively high, with a minimum correlation of 0.72 for subject 2 (motor execution and ipsilateral side). Typically, the closest SS channels were most similar to the reference LS channel, and the MW band was less influenced by the motor-execution tasks than the HR band. The global PCA model showed consistently lower correlations than the contralateral condition, but still achieved values in the same order of magnitude (i.e., r≥0.74).

Figure 5 shows that the channels most similar to the reference LS channel are ${\mathrm{SS}}_{1}$, ${\mathrm{SS}}_{3}$, ${\mathrm{SS}}_{5}$, and ${\mathrm{SS}}_{7}$, which are located closest and symmetrically opposite to the LS channel. Correlations between SS channels follow a symmetrical pattern with opposite SS channels showing the highest correlation, i.e., the SS channel pairs ${\mathrm{SS}}_{1}$ versus ${\mathrm{SS}}_{5}$, ${\mathrm{SS}}_{2}$ versus ${\mathrm{SS}}_{6}$, ${\mathrm{SS}}_{3}$ versus ${\mathrm{SS}}_{7}$, and ${\mathrm{SS}}_{4}$ versus ${\mathrm{SS}}_{8}$. While time lags among the SS channels show positive and negative values, the LS channel consistently lagged behind the SS channels in the range of −0.19 to −0.79  s for the MW band. The correlation coefficients for the MW band were larger than for the HR band, especially when the signals are phase shifted (lower tables in Fig. 5).

### 3.2 Simulation of Hemodynamic Response Time Series

In this section, we report on the effectiveness of five GLM regression approaches to remove physiological artifacts from simulated measurements. For each subject, a total of 54 HRs were simulated, which were overlaid on resting-state measurements of a reference LS channel over the left primary motor cortex. All regression approaches were applied on the same time courses, but used different sets of SS signals (all obtained from resting-state SS channels). For each subject, a t-value using a separate GLM model was calculated, as well as correlation (r) and RMSE between the recovered and the ideal HR. Normalized t-values were obtained from the ratio of every regression method and the reference method ${\mathrm{GLM}}^{\mathrm{SS}}$. A larger value indicates either an HR with a stronger amplitude and/or a less noisy signal in reference to ${\mathrm{GLM}}^{\mathrm{SS}}$.

**Fig. 6:**
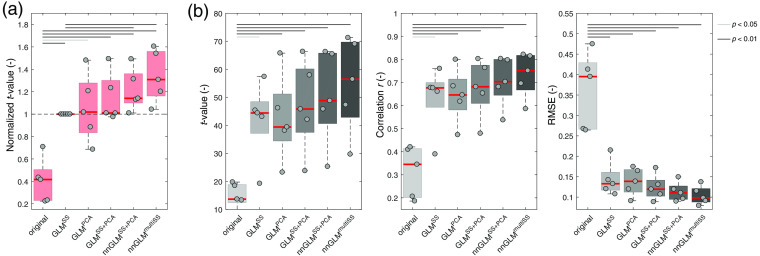
Effectiveness of brain activity estimates for resting-state simulations. Box plots show the (a) normalized and (b) absolute values. Normalized t-values were obtained as the ratio of t-values between each GLM regression and the ${\mathrm{GLM}}^{\mathrm{SS}}$ approach (being the most common of the used methods). t-values were obtained after run-wise analysis of the entire time course by means of a GLM. The gray dots and red lines indicate individual subjects (subjects 1 to 5 in horizontal order) and the group median values, respectively. Results are shown for the simulated time courses, where artificial HRs were superimposed on resting-state measurements. A total of 270 trials were evaluated.

All GLM regression approaches enabled the more effective estimation of brain estimates in comparison to the original LS signal (i.e., no regression applied), expressed as significant differences for all metrics in Fig. 6. No significant difference between ${\mathrm{GLM}}^{\mathrm{PCA}}$ and ${\mathrm{GLM}}^{\mathrm{SS}}$ was observed. There was no difference in normalized t-values despite some intersubject variability: subjects 2 and 3 benefited especially from the global regressor and subjects 4 and 5 from the local regressor. The improvement in normalized t-values for ${\mathrm{GLM}}^{\mathrm{SS}+\mathrm{PCA}}$ was small, but overall its performance was equal (subjects 1, 4, and 5) or better (subjects 2 and 3) than the benchmark ${\mathrm{GLM}}^{\mathrm{SS}}$ method. ${\mathrm{GLM}}^{\mathrm{SS}+\mathrm{PCA}}$ benefited from local and global regressors by combining the regressor signals of the two simpler methods ${\mathrm{GLM}}^{\mathrm{SS}}$ than ${\mathrm{GLM}}^{\mathrm{PCA}}$.

The additional inclusion of MW oscillations in $nn{\mathrm{GLM}}^{\mathrm{SS}+\mathrm{PCA}}$ and $nn{\mathrm{GLM}}^{\mathrm{multiSS}}$ further improved the estimates of brain activity. In particular, both methods lead to significant improvements in normalized t-values with a median change of +14% and +31%, respectively, in comparison to ${\mathrm{GLM}}^{\mathrm{SS}}$. Although there was no significant difference of absolute values in Fig. 6(b), a trend toward the higher effectiveness of the same approaches is visible.

### 3.3 Motor-Execution Task

**Fig. 7:**
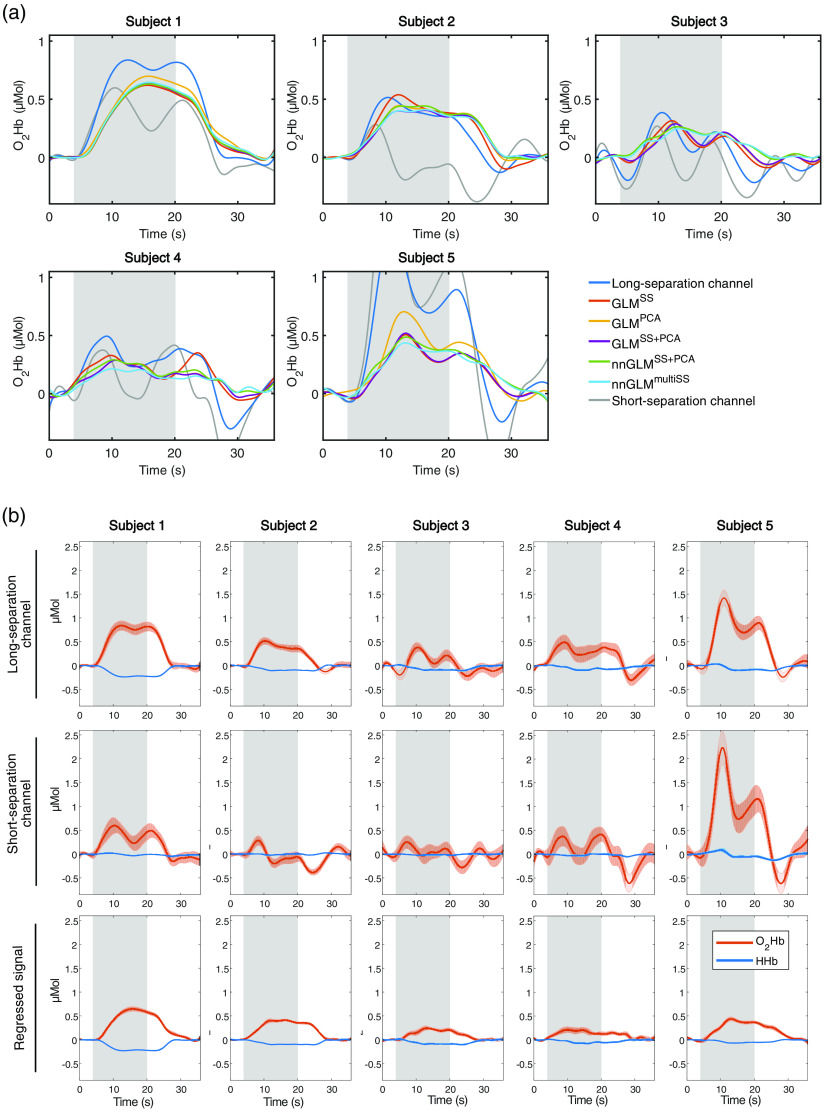
Block average time traces for the motor-execution experiment. Block-averaged $O_{2}\mathrm{Hb}$ and HHb responses from all hand-grasping trials are shown for a time segment of 34 s for each of the five subjects. Gray bars indicate the 16-s hand-grasping task, white bars indicate rest. (a) Averages ($O_{2}\mathrm{Hb}$) for the original LS channel (located over primary motor cortex), the five investigated GLM approaches, and the closest SS channel (${\mathrm{SS}}_{3}$) are shown. (b) Close-up plots of relative concentration changes of $O_{2}\mathrm{Hb}$ and HHb (red and blue lines with median value and median absolute deviation) for the LS channel, the SS channel (${\mathrm{SS}}_{3}$) and the regressed signal using $nn{\mathrm{GLM}}^{\mathrm{multiSS}}$.

The block averages of relative concentration changes for the five subjects in Fig. 7 illustrate the influence of the short-channel regression on the brain estimates. The signals were averaged over all hand-grasping trials obtained during the two runs (when applicable). All subjects showed an HR in the reference LS channel (placed over the left primary motor cortex) during the right-hand grasping task, and a shape more similar to the ideal HR was obtained after short-channel regression. The SS channels (${\mathrm{SS}}_{3}$ was used by way of illustration) exhibited a relatively flat curve for subjects 2 to 4 and showed a larger activation for subjects 1 and 5 as an indicator of strong systemic activity.

**Fig. 8:**
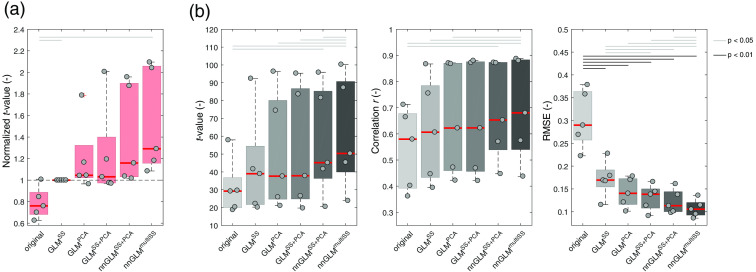
Effectiveness of brain activity estimates for the hand-grasping experiment. Box plots show (a) normalized and (b) absolute values. Normalized t-values were obtained as the ratio of t-values between each GLM regression and the ${\mathrm{GLM}}^{\mathrm{SS}}$ approach (being the most common of the used methods). t-values were obtained after run-wise analysis of the entire time course by means of a GLM. The gray dots and red lines indicate individual subjects (subjects 1 to 5 in horizontal order) and the group median values, respectively. Results are shown for the simulated time courses, where artificial HRs were superimposed on resting-state measurements. A total of 180 trials were evaluated.

All GLM methods achieved better estimates of brain activity than the unregressed original LS signal in Fig. 8. However, for the motor-execution experiment, the improvements in t-value and correlation were less pronounced than for the simulations (Sec. 3.2), and mainly the RMSE metric showed a statistically significant improvement. For subject 4, applying short-channel regression led to no or small changes in t-value while the RMSE strongly decreased. This presumably is a consequence of the strong systemic activation and an accompanying decrease of signal amplitude after regression. No superiority of ${\mathrm{GLM}}^{\mathrm{PCA}}$ over ${\mathrm{GLM}}^{\mathrm{SS}}$ was observed, with a slight, nonsignificant trend toward ${\mathrm{GLM}}^{\mathrm{PCA}}$. Although the median normalized t-values for ${\mathrm{GLM}}^{\mathrm{PCA}}$ and ${\mathrm{GLM}}^{\mathrm{SS}}$ were nearly identical on a group level, there were distinct intersubject differences, e.g., with subject 2 profiting strongly from the global regressor (+79% for ${\mathrm{GLM}}^{\mathrm{PCA}}$). ${\mathrm{GLM}}^{\mathrm{SS}+\mathrm{GLM}}$ performed similarly to ${\mathrm{GLM}}^{\mathrm{PCA}}$. No significant difference in t-values was observed but significantly lower RMSE.

The additional inclusion of MW oscillations in $nn{\mathrm{GLM}}^{\mathrm{SS}+\mathrm{PCA}}$ led to better brain activity estimates in comparison to the ${\mathrm{GLM}}^{\mathrm{SS}}$ benchmark, expressed as a 15% median improvement in normalized t-value and a significant decrease in RMSE. $nn{\mathrm{GLM}}^{\mathrm{multiSS}}$ returned the best estimated brain activity, with an overall improvement in normalized t-value of 29% and significant changes in correlation and RMSE compared to ${\mathrm{GLM}}^{\mathrm{SS}}$. Both methods were more effective on single-subject level, showing an improvement in all metrics observable for every subject.

## 4 Discussion

A better understanding of systemic hemodynamics is crucial for the correct interpretation of fNIRS measurements and, thus, the future usability of fNIRS for research and clinical applications. While short-channel regression has been identified as an important step in making fNIRS technology more applicable, the important question of which systemic signals to regress remains insufficiently addressed. In this work, we investigated in detail the fNIRS signals from 8 SS channels (8-mm source–detector separation) over sensorimotor brain areas by comparing them with a reference LS signal (separation: 30 mm, located over the left primary motor cortex) during resting-state and a hand-grasping experiment. We evaluated five GLM regression approaches that made use of different regressor signals, which were selected from literature and physiological assumptions. We proposed two regression approaches based on non-negative least squares to include additional spatial and temporal information of the scalp (i.e., multiple scalp regressors and their phase-shifted MW signals) compared to state-of-the-art approaches. We show the improved effectiveness to remove physiological noise, thereby shedding light on the optimal selection of scalp regressors to obtain better estimates of functional brain activation.

### 4.1 Heterogeneous versus Homogeneous Scalp Hemodynamics

With respect to the incongruent assumptions on the spatial distribution of scalp hemodynamics (heterogeneous^9^^,^^17^^,^^28^^,^^31^^–^^38^ versus homogeneous^22^^,^^26^^,^^27^^,^^29^^,^^30^) reported in literature for fNIRS experiments, we found evidence for a location-specific behavior of the scalp hemodynamics. This supports the hypothesis of heterogeneity. However, we also observed a global spatial distribution with close-to-homogeneous characteristics, mainly when analyzing the resting-state measurements. In particular, we showed that the hemodynamics are more similar for close and symmetrical (interhemispheric) scalp regions and that the propagation of the hemodynamics is delayed between regions. MW oscillations were delayed between different SS channels and in reference to the reference LS channel, and correlations improved after applying phase shifting. From these findings, we conclude that scalp hemodynamics at different locations can be interpreted as a superposition of the same physiologically originating signals (e.g., MWs, task-evoked systemic activation), however, with slight variations of the same signals at every location due to time lags, nonstationarities and nonlinearities.

Our results are in agreement with recent publications investigating scalp hemodynamics. We confirm the findings of Gagnon et al.^33^ that an LS channel over the contralateral motor region is better correlated to close than more distant SS channels, however, the phenomenon was less prominent in our case. Zhang et al.^36^ also found, for a resting-state measurement with short channels distributed over the whole head, higher correlations for close and symmetrical short channels among themselves. In particular, they showed a more homogeneous behavior for the MW band than for the whole-frequency band, similar to what we observed for the MW and HR bands. They further observed time lags of 0.35 and 0.83 s for close and symmetrical short channels in the MW band, which is in the same range as our findings (0.19 to 0.79 s). Furthermore, for the local and the symmetrical configurations in the MW band, they measured relatively large correlation values (r≥∼0.78), being in the same range as our correlations (r≥0.75).

Based on the evidence from this work and in line with literature, it is hard to deny the observed heterogeneity in scalp hemodynamics over sensorimotor brain regions. However, this clearly comes on top of an underlying homogeneous behavior, as suggested by Sato et al.,^29^ and depending on the intended application, this simplified assumption of homogeneity might be a reasonable compromise. Since there is no strict threshold for when homogeneity ends and heterogeneity begins, similar observations may lead to different conclusions on the spatial distribution of scalp hemodynamics. We believe that the incongruent assumptions of homogeneity and heterogeneity can be explained by different experimental conditions, research hypotheses, and evaluation approaches. Multiple factors may further influence the observed spatial patterns and should be investigated in more detail, e.g., the used fNIRS instrumentation, the optode locations (frontal, temporal, or occipital regions), the experimental protocol (resting state versus functional task), or the selection of evaluation metrics.

In the first part of this work, we conclusively demonstrated that scalp hemodynamics follow a heterogeneous distribution. While this was proposed before for baseline/resting-state measurements for the entire head on a larger scale^36^ or the contralateral sensorimotor regions only,^33^ we extended the notion of heterogeneity to left and right sensorimotor areas, and different experimental protocols (resting state and motor execution) and frequency bands (HR and MW bands). We showed that heterogeneity is also observable in the MW oscillations and that phase shifting increased correlations but also that the heterogeneous pattern was manifested less prominently in the MW than the HR band. These findings underline the importance of taking into account heterogeneous scalp hemodynamics during short-channel regression in order to remove physiological noise as thoroughly as possible.

### 4.2 Physiological Explanations for Scalp Heterogeneities

The observed interhemispheric symmetry between SS channels is consistent with the literature.^36^^,^^58^ We assume this observation to be a consequence of location-specific characteristics of the underlying vessels. In particular, the scalp is predominantly supplied from the left and right external carotid arteries (except parts of the frontal regions, which are supplied from the internal carotid arteries), which branch into multiple larger and smaller arteries to supply different scalp areas. The scalp regions above the sensorimotor cortices are supplied from a tree-like structure consisting of the superficial temporal arteries and its smaller branches. It is possible that symmetrical interhemispheric arteries and arterioles have more similar path lengths and sympathetic mediation^12^ than closer vessels on the same hemisphere, explaining the location-specific perfusion and the reported symmetry. A similar structure is observed for the venous vessels as well. Furthermore, Yücel et al.^15^ showed that the amplitudes of SS signals (in the MW band) can differ across the head and explained this by the anatomy of the underlying vessels and the relative positioning of the optodes: If a larger artery is located below an optode, stronger MW oscillations are expected. We preclude other factors that may lead to the reported symmetry: (1) It is unlikely that significant cerebral activation was co-registered with a 7-mm SS channel (the expected sensitivity to the brain is <1%^24^). (2) The influence of emissary veins, which connect scalp and cerebral venous tissue for pressure equalization, is not entirely understood, but it was hypothesized that their diameter is too small to generate strong hemodynamic changes.^12^ (3) Instrumentation noise is not expected to generate symmetrical patterns.

The exact mechanism and function of MW are still subject to discussion, but it is known that they are spontaneous oscillations in arterial pressure linked to the baroreceptor loop.^14^^,^^59^^,^^60^ The baroreceptor loop is a homeostatic mechanism responsible for the regulation of the blood pressure. Thus, MW is a global phenomenon that is observed all over the body. It is important to note that “global” does not necessarily imply homogeneity in the entire body or particularly the brain and scalp. Therefore, our observation of heterogeneity in scalp MW oscillations is not in disagreement with the existing knowledge about MW, most of all since the heterogeneity was only weakly manifested and comes on top of a global close-to-homogeneous distribution. This observation may again be a consequence of slight variations in the anatomy and innervation of the measured vessels. Other physiological contributors than MW exist that may generate signal heterogeneities in the MW band, but whose contribution cannot be separated from MW in this work. (1) Vasomotion designates spontaneous changes in the vasomotor tone and generates similarly strong oscillations than MW.^59^^,^^60^ The exact interplay between MW and vasomotion remains unclear,^12^^,^^15^ and it is a challenging task to separate the two phenomena. (2) Evoked systemic activation may lead to spurious activation in the MW-frequency band.^19^

In our measurements, we observed that the reference LS signal was lagging behind the SS signals, similarly to what was reported in Kirilina et al.^12^ This is an interesting observation since the LS signal is a superposition of signals from superficial and deep-layer origin. A constant (negative) time lag between the LS and SS channels implies that either the systemic activity is more delayed in the cerebral compartment compared to the scalp, or spontaneous signals (spontaneous neural activity or spontaneous vasomotion) are co-registered.^28^^,^^61^^,^^62^ Kirilina et al.^12^ hypothesized that the time lag between cerebral and extracerebral tissue is a consequence of different vascular path lengths and possible delayed sympathetic mediation between the deep and superficial layers. We support this hypothesis and want to emphasize that the brain and scalp are supplied by different arteries (i.e., from the internal and external carotid, respectively), which already branch relatively low at the level of the neck (i.e., carotid sinus). Since these time lags between tissue layers reduce the effectiveness of the short-channel regression, it will be important to further elucidate the interconnection between cerebral and extracerebral systemic signals and investigate further ways to assess them as well as to include them as regressors.

### 4.3 Effect of Heterogeneous Scalp Hemodynamics on Short-Channel Regression

The effect of five different scalp regressors on the outcome of short-channel regression was investigated. All five GLM approaches significantly improved the signal quality of the recovered time series by reducing the physiological noise. This was expressed as increase in t-values and Pearson’s correlation (r) and a decrease in RMSE, when comparing the recovered with a modeled HR. The ability to improve signal quality of fNIRS measurements based on short-channel regression is undisputed in the community^2^ and was confirmed with this work.

No advantage or disadvantage for the use of a global regressor (${\mathrm{GLM}}^{\mathrm{PCA}}$) over the conventional local regressor (${\mathrm{GLM}}^{\mathrm{SS}}$) was observed for our experimental conditions. Similarly, Tian et al.^28^ were not able to show the superiority of a local over a global regressor. However, these results are different from Erdoğan et al.^17^ and Goodwin et al.,^37^ where the global regressors performed worse. These presumably contradicting findings again may be explained by different experimental conditions, and we believe that particularly the way that the global regressor is calculated has a strong influence on the results. The small difference between ${\mathrm{GLM}}^{\mathrm{PCA}}$ and ${\mathrm{GLM}}^{\mathrm{SS}}$ is consistent with our observations that the scalp hemodynamics shows a global distribution close to homogeneity over sensorimotor areas.

We confirmed that the combined use of a local and global regressor (${\mathrm{GLM}}^{\mathrm{SS}+\mathrm{PCA}}$) led to improved effectiveness of removing systemic artifacts in comparison to ${\mathrm{GLM}}^{\mathrm{SS}}$ and ${\mathrm{GLM}}^{\mathrm{PCA}}$.^17^ The improved performance can be explained by the physiological assumption that the LS fNIRS signal is a combination of extracerebral and cerebral components, and while the local regressor better covers the superficial component, the global regressor may introduce additional information more similar to the cerebral signal. Therefore, it is suggested whenever possible to consider the multilayered behavior of fNIRS signals and include both local and global regressors.

Building on the finding of delayed MW oscillations between different scalp regions and between cerebral and superficial compartments, two of the five regression approaches attempted to specifically consider temporal heterogeneity of the fNIRS signals. By including the phase-shifted MW signal from the scalp as a separate regressor, we proposed a way to independently consider one of the strongest contributors to the degradation of fNIRS measurements and to take into account their delayed oscillations.^19^^,^^28^ Non-negative least squares enabled the inclusion of additional information by finding a sparse solution to the linear equation model by setting the weights of “unuseful” regressors to zero, thereby, inherently applying a channel selection procedure. Both introduced approaches ($nn{\mathrm{GLM}}^{\mathrm{SS}+\mathrm{PCA}}$ and $nn{\mathrm{GLM}}^{\mathrm{multiSS}}$) achieved higher t-values compared to the other three, state-of-the-art approaches. It was observed that the effectiveness of the different regressors are subject-dependent, e.g., subject 3 improved strongly when the MW were time shifted and subject 2 benefited from the PCA regressor in the motor-execution experiments. Interestingly, the results even improved for the subjects who showed strong activations already before short-channel regression (i.e., subjects 1 and 2). The benefit of using phase-shifted regressor signals confirms the results of Tian et al.^28^ and von Lühmann et al.,^63^ who found improved regression outcomes when a separate adaptive filter for MW or a phase-shifting approach based on canonical correlation analysis (CCA) was applied.

Our findings highlight that short-channel regression can be further improved when making more sophisticated assumptions based on human physiology. This may be important for brain research when a more exact estimation of the location and magnitude of the HR is necessary, but also for single-trial applications (e.g., BCI) requiring “clean” signals to minimize false positives and false negatives.

### 4.4 Limitations and Outlook

This study is limited due to the relatively low number of participants. Nevertheless, a comprehensive view of the generalizability of our results is obtained. The subjects had very different signal contents: subjects 1 and 2 had strong HRs, subjects 3 and 5 had large MW amplitudes, and subject 5 had very strong task-evoked systemic activation. Nevertheless, the two nnGLM approaches improved the results for all subjects and conditions, implying that no negative effect from their application should be expected.

The MW-bandpass filtered signal may not solely include MW oscillations, but also contributions from task-evoked systemic activation and the HR (contained in the LS channel). Therefore, there is a risk that removing specific frequencies may alter the magnitude or slope of the HRs after regression.^32^ This risk is small for the investigated protocol with block durations of 30 to 34 s but will increase when shorter block durations would be used due to more overlapping frequency contents between functional activation and MW.^64^ Furthermore, the computational power required for the nnGLM approaches is higher, mainly due to the bandpass filtering and phase shifting of the regressor channels. We proposed the application of non-negative least squares also for the regression of other biosignals (e.g., blood pressure^10^^,^^65^ and peripheral fNIRS measurements^38^).

In the simulation part of this work, we did not assume any systemic task-evoked activation, which minimizes the possibility to overcompensate systemic signals. It is known that during a functional task, the autonomic nervous system activity is increased,^66^^,^^67^ which leads to a task-evoked reaction in the SS channels.^13^^,^^34^ The strength of systemic activation is influenced by task-evoked changes in the mean arterial blood pressure,^8^^,^^68^ and thus different protocols may lead to different scalp artifact patterns. Also, different sets of global/local regressor signals and ways of calculating them could be considered and could alter the brain activity estimates. For example, a global regressor could be combined with local scalp signals, which are obtained from the residuals after regression of the global signal from each SS signal.

In this study, we deliberately used a simple regression method and performed the regression offline on the entire dataset. Future studies should compare our findings with other approaches proposed in the literature, such as autoregressive models,^47^ Bayesian filtering,^69^ or the recently proposed temporally embedded CCA (tCCA).^63^ The tCCA approach also considers latencies in the regressor signals and may offer an alternative to the proposed selection of the optimal delay based on least-squares minimization between LS and SS signals. For real-time applications, we suggest to elaborate on the compatibility of the proposed approach (non-negative weight estimation) with algorithms that inherently consider phase shifts by design,^18^^,^^27^^,^^28^ e.g., adaptive filters.^70^ Alternatively, the phase shifts of the MW signals could be calculated for a baseline measurement^71^ when applied with a sliding window approach.^30^ Real-time algorithms may further improve regression performance because of the nonstationary and nonlinear nature of SS signals.^18^^,^^72^

The hardware employed provided SS channels next to the sources but not next to the detectors. Results may have looked different with additional SS channels located at the detectors.^34^^,^^73^ Therefore, it will be important for future applications to apply hardware that captures scalp hemodynamics at both source and detector sites.^74^^,^^75^

## 5 Conclusion

With this work, we aimed to shed light on the behavior of scalp hemodynamics over the sensorimotor cortex and the influence of scalp regressors on short-channel regression. The better understanding of the systemic physiology enhances the estimates of functional cerebral activation, and is an important step in promoting routine application of fNIRS, especially at the individual level as required in clinical applications. We conclude that

1. The scalp hemodynamics follows a heterogeneous and frequency-specific behavior. These heterogeneities are superimposed on a global, close-to-homogeneous distribution.
2. The introduction of an adapted GLM approach using non-negative least squares enabled the inclusion of multiple regressors with a reduced risk of overfitting compared to state-of-the-art methods. We tested five regressor combinations and found that better performance was achieved when assuming heterogeneous scalp hemodynamics. In particular, we showed the benefit of considering delayed MWs in the regression to compensate for their phase-shifted oscillations between different scalp regions and compartments (cerebral versus superficial).

With this work, we highlighted the importance of applying short-channel regression and present a way to unite the multilayered behavior of systemic signals in a regression algorithm. We proposed an easy-to-implement short-channel regression method based on GLM and showed the benefit of including multichannel and multifrequency information. We encourage all future fNIRS studies to concomitantly capture SS channels to reduce the influence of systemic physiology.

## 6 Appendix: Single-Trial Evaluation

A CNR metric was calculated to investigate the effectiveness in improving single-trial estimation of brain activity. A high CNR value gives an indication on the ability of single-trial classification, for example in the frame of a BCI.

The trial-based CNR metric was established in reference to Saager et al.,^73^ where for every trial, a specific curve (e.g., a skewed Gaussian curve^37^^,^^73^) was fitted. We adapted the method by fitting an artificial HR into the segmented trials instead of a Gaussian curve. Fitting an artificial HR has the advantage that the ideal shape has a stronger influence on the results than more simplistic functions or frequency-based CNR approaches.^27^^,^^44^^,^^76^ For every trial, we iteratively minimized (MATLAB: fminsearchbnd) the RMSE between the artificial response and a selected segment (i.e., from trial onset until the next intertrial end).^77^ The simulated HR was created by convolution of the boxcar and the double gamma function.^78^ During the optimization procedure, amplitude of response, delay of response, delay of undershoot, and a constant offset of the HR function were varied, whereas all other variables of the double gamma kernel were kept constant. To preserve realistic shapes, we restricted the boundaries for the delay of response and the delay of undershoot to: 4 to 10 s and 10 to 20 s.^77^ For every trial, three trial-wise metrics were obtained:^79^ (i) the CNR by calculating the ratio of the fitted artificial response’s amplitude ($S^{i}$) divided by the ${\mathrm{RMSE}}^{i}$ (${\mathrm{CNR}}^{i}=S^{i}/{\mathrm{RMSE}}^{i}$), (ii) Pearson’s correlation coefficient ($r^{i}$) between artificial and recovered responses, and (iii) ${\mathrm{RMSE}}^{i}$ of the segment’s residuals. A higher single-trial CNR increases the chance to flag a trial as active,^37^ and, prospectively, implies a reduction of needed repetitions to detect significant differences between tasks, or a more reliable classification during BCI applications. A CNR value of ≥2.5 was empirically found to have a probability higher than 95% that the recovered signal contains an activation, as calculated with a one-sided t-test on randomized tasks during rest condition.

**Fig. 9:**
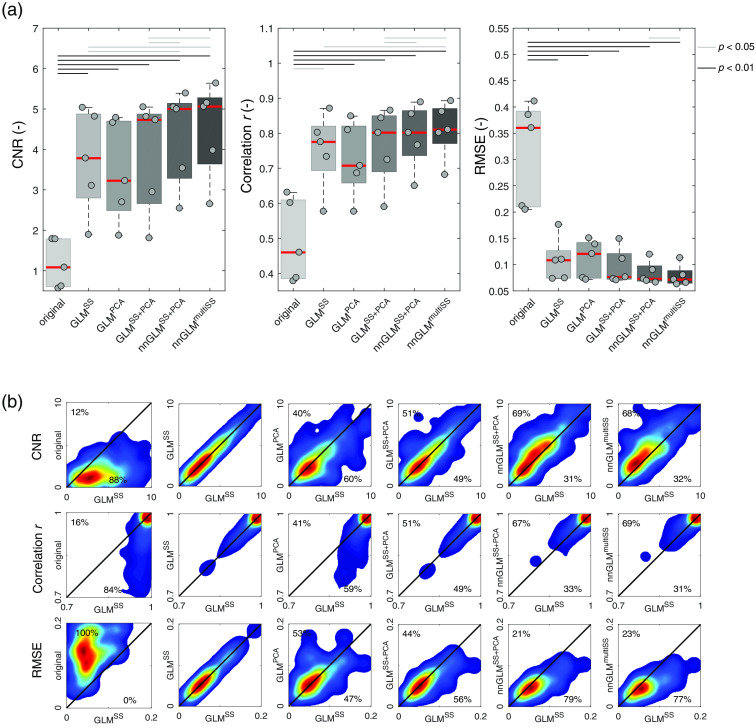
CNR for resting-state simulations. (a) Box plots show absolute values of CNR, correlation, and RMSE. The gray dots and red lines indicate individual subjects (subjects 1 to 5 in horizontal order) and the group median values, respectively. (b) CNR, RMSE, and correlation (r) values were obtained for each trial between an iteratively fitted artificial HR and the actual signal, and the results for each GLM regression approach (y axis) are depicted with respect to the reference ${\mathrm{GLM}}^{\mathrm{SS}}$ method (x axis). Results are obtained for a total of n=270 simulated HRs.

**Fig. 10:**
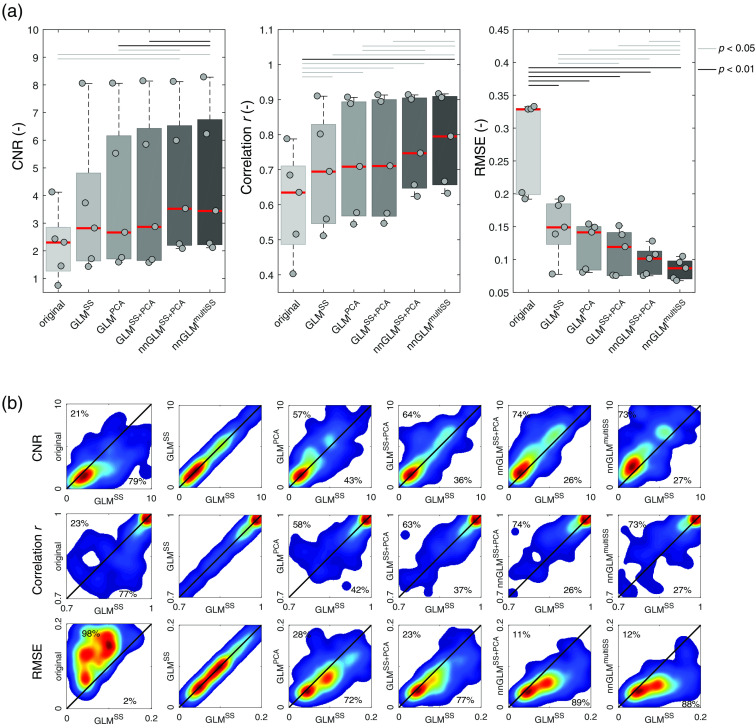
CNR for the hand-grasping experiment. (a) Box plots show absolute values of CNR, correlation, and RMSE. The gray dots and red lines indicate individual subjects (subjects 1 to 5 in horizontal order) and the group median values, respectively. (b) CNR, RMSE, and correlation (r) values were obtained for each trial between an iteratively fitted artificial HR and the actual signal, and the results for each GLM regression approach (y axis) are depicted with respect to the reference ${\mathrm{GLM}}^{\mathrm{SS}}$ method (x axis). Results are obtained for a total of n=180 hand-grasping trials.

Results in Figs. 9(a) and 10(a) indicate a similar performance of the CNR metric as for the run-wise t-values from the main body of this work. All short-channel regression methods achieved higher CNR values compared to the original case. ${\mathrm{GLM}}^{\mathrm{SS}}$ and ${\mathrm{GLM}}^{\mathrm{PCA}}$ did not show a significant difference. The methods $nn{\mathrm{GLM}}^{\mathrm{SS}+\mathrm{PCA}}$ and $nn{\mathrm{GLM}}^{\mathrm{multiSS}}$ using non-negative least squares and including separately MW signals performed best.

In Figs. 9(b) and 10(b), scatter plots indicate the ability to improve single-trial estimation of brain activity in comparison to the ${\mathrm{GLM}}^{\mathrm{SS}}$ method. The percentages indicate the number of trials that achieved a higher (upper triangle) or smaller (lower triangle) value compared to ${\mathrm{GLM}}^{\mathrm{SS}}$. Here, the previous findings are confirmed, with all regression methods outperforming the original signal, and the non-negative GLM methods achieving best results.
